# The relationship between fall and loneliness among older people in China: the mediating role of personality trait

**DOI:** 10.3389/fpsyt.2023.1204544

**Published:** 2023-08-08

**Authors:** Luo Yuan, Wu Yibo, Deng Yuqian, Ran Haiye, Liu Jiaxin, Zhao Liping

**Affiliations:** ^1^School of Nursing, Capital Medical University, Beijing, China; ^2^School of Public Health, Peking University, Beijing, China; ^3^Xiang Ya Nursing School, Central South University, Changsha, Hunan Province, China; ^4^Xiang Ya Second Hospital of Central South University, Changsha, Hunan Province, China

**Keywords:** loneliness, social isolation, accidental fall, older people, big five personality, mediating effect

## Abstract

**Aims:**

We aimed to explore the role of personality traits between fall and loneliness.

**Methods:**

A questionnaire survey was used to investigate falls, the big five personality traits, and loneliness among older people (≥ 60 years old) in China mainland.

**Results:**

A total of 4,289 older people participated in the survey. There are significant differences in age, marital status, education level, residence, solitariness, and fall in relation to loneliness among older people. Falls, especially when they occurred one time increase the loneliness of older people. Agreeableness, conscientiousness, and neuroticism were significant mediating effects between falls and loneliness.

**Conclusion:**

This study implied that agreeableness, conscientiousness, and neuroticism were meditating factors between falls and loneliness. In the future, we should consider the big five personality traits more to understand loneliness and offer older people interventions for reducing their loneliness. The study design was cross-sectional, so the temporal precedence of mediators and causality could not be tested. Because the data were collected retrospectively, current loneliness is likely to have confounding effects on retrospective recall.

## 1. Introduction

In general, loneliness is the discrepancy between a person’s preferred and actual level of social contact. Loneliness reflects a subjective feeling of the absence of desired affection and closeness from close family members, intimate friends, or someone significant (1). With the accelerating aging and increasing life expectancy in China, loneliness is growing among older people, especially older people with poor health (2). In China, for example, a national survey found that 29.6% of older people (≥ 60 years old) reported that they ‘often felt lonely’ (3). During the pandemic, especially, 34.2% of older people suffered from loneliness, of which 15.5% were severely lonely (4). Many studies showed that loneliness is highly related to the health status of older people. In a large sample survey, loneliness strongly indicated the development of pain, fatigue, and depression as well as the cluster of all three symptoms several years later (5). For mental health, loneliness was longitudinally associated with posttraumatic stress symptoms, and there might be a bidirectional predictive relationship (6, 7).

In China, as the aging process accelerates, fall is still a serious problem. It was reported that the annual fall rate among Chinese older people ranged from 11 to 34% (8); 54.95 per 1,000 injuries would be fall-related injuries (9), and unintentional falls mortality rose from 7.65 to 8.03 per 100,000 people between 2006 and 2016 (10). According to a survey, older people who have experienced a fall would reduce social participation actively (11). After older people experienced a fall, they would make activity restrictions and gradually make subsequent social isolation (12). This could further increase feelings of loneliness. Fall was associated with loneliness among older people, which was common for those living alone (13, 14). A longitudinal study showed that both low social contact and living alone among older people were highly associated with self-reported falls even after controlling for sociodemographic, health, and lifestyle variables (15). According to the German Aging Survey, any fall in the past 12 months was associated with increased odds of loneliness among older adults. A higher level of loneliness showed in more times fall occurred (16). A systematic review implied the association between falls and loneliness among older people (17). Therefore, we need to explore the relationship between fall and loneliness further.

Since the 1990s, the Big Five personality has been a hot research topic, which consists of extraversion, agreeableness, conscientiousness, neuroticism, and openness (18, 19). While the Big Five personality may be regarded as an important model in personality studies, it is not an integrative model (19). Each personality trait has a unique impact on psychology and behavior. A systematic review showed that different personality traits had different attitudes to falls among older people (20). That was important for the successful implementation of fall-prevention programs. A study indicated that the relationship between loneliness and personality was largely explained by its relationship with neuroticism (21). Neuroticism was positively associated with indoor falls and recurrent outdoor falls (22). Moreover, it was proved that agreeableness among older people was negatively associated with loneliness (23, 24). Fallen older people feel so lonely that they reduce social contact. If they are unwilling to seek help due to their personality traits, they will experience more loneliness as a result (11, 12, 25). Therefore, it is important that the Big Five personality traits be studied to assist in understanding loneliness and offer the interventions for reducing loneliness among older people.

In summary, fallen older people are more likely to feel lonely. At present, although the relationships among the variables of fall, loneliness, and personality trait have been examined separately, there is a lack of studies to explore the relationship between the three. Hence, this study aims to explore the mediating role of personality traits between falls and loneliness among older people in China.

## 2. Methods

### 2.1. Participants and procedures

A multi-stage sampling method was selected from 20 June to 31 August in the “Psychology and Behavior Investigation of Chinese Residents in 2022, PBICR.” In this cross-sectional survey study, the participants were from 23 provinces, 5 autonomous regions, and 4 municipalities directly under the central government in China. Training investigators distributed the questionnaires to participants face-to-face and one-on-one (26). This study has been officially registered in the China Clinical Trial Registry (Registration No. ChiCTR2200061046). A total of 31,480 questionnaires were distributed. The inclusion criteria:(1) The participants had the nationality of the People’s Republic of China; (2) they voluntarily participated in the study and signed an informed consent form; and (3) they understood each item of the questionnaire and completed the questionnaire on their own or with the help of an investigator. The exclusion criteria: (1) The participants were confused, mentally abnormal, or have cognitive impairment; (2) they were currently participating in other similar studies. The excluding invalid questionnaires: (1) filling time ≤ 100 s; (2) conflicting answers between entries; and (3) incompletely filled. Finally, 30,505 valid questionnaires were collected, with an effective rate of 96.9%.

In our study, older people were selected. The age of participants must be equal to or more than 60 years old. Finally, 4,289 older people were enrolled in this study.

### 2.2. Measures

#### 2.2.1. Sociodemographic characteristics

The questionnaire also contained questions related to participants’ sociodemographic characteristics, including age, gender, marital status, education level, monthly family income, residence, and solitariness.

#### 2.2.2. Fall

Falls are defined as sudden, uncontrollable changes in body posture that result in an individual falling to the ground or low flat surfaces, which can result in serious injuries, unconsciousness, or seizures (9). In this study, falls are divided into three categories: (1) No fall; (2) A single fall means that the fall frequency is once; and (3) Multiple falls mean that fall frequency is more than once. The survey explored the number of falls in the last 3 months.

#### 2.2.3. Personality

The Ten-Item Big Five Personality Inventory (BFI-10) (27, 28) was selected to investigate personality. It is a very short measure of the Big Five personality traits (extraversion, agreeableness, conscientiousness, neuroticism, and openness). Each personality dimension is measured by two items. The scale is adopted on a 5-point Likert scale ranging from one (disagree strongly) to five (agree strongly). Items 1, 3, 4, 5, and 7 are reverse scoring, and items 2, 6, 8, 9, and 10 are forward scoring. Higher scores indicate higher levels of a given personality trait. The Cronbach’s α of the BFI-10 in our study was 0.600.

#### 2.2.4. Loneliness

Loneliness was measured using the Three-Item Loneliness Scale (T-ILS) (29). The items for the T-ILS were “How often do you feel left out?,” “How often do you feel isolated from others?,” and “How often do you feel that you lack companionship?” The scale is adopted on a 3-point Likert scale (1 = hardly ever, 2 = some of the time, and 3 = often). The score range of the T-ILS is thus three to nine. Loneliness becomes more apparent as the score rises. The T-ILS was originally derived from the UCLA Loneliness Scale, which was used for older people (30). In many studies, the T-ILS is increasingly being used for older people (31, 32). The Cronbach’s *α* of the T-ILS in our study was 0.849.

### 2.3. Statistical analysis

SPSS 26.0 was used to make statistical analysis. Descriptive statistics of sociodemographic characteristics and variables of interest were reported. Frequency and percentage were used to describe categorical variables. Continuous variables were reported on the mean (*M*) and standard deviation (SD). One-way ANOVA test and independent t-test were used to examine the differences in loneliness. *p* < 0.05 was considered statistically significant. Further, we used the *post hoc* test for significant difference factors by the statistics method of LSD. Spearman analyses were performed to examine whether there is a correlation between the big five personality traits and falls. Pearson correlation analyses were performed to examine whether there is a correlation between the big five personality and loneliness. Multiple linear regression analysis was conducted to further explore the associated factors which had statistical significance in univariate analysis.

The macro-program PROCESS 3.4 was selected to examine the mediation of the big five personality traits between fall and loneliness (33, 34). Model 4 was used to test the direct, indirect, and total effects based on 5,000 bootstrapped samples. The mediating effect was significant if the 95% bias-corrected confidence interval did not include zero.

## 3. Results

### 3.1. Common method biases tests

For this study, subjectivity was excluded as much as possible in order to avoid common method bias. The samples were collected nationwide and anonymously. In the meantime, the exploratory factor analysis method was used to test the common method bias. The results revealed three factors with an eigenvalue greater than one, and the total variation explained by the first factor was 22.303%, which was far lower than the critical value of 40%. Thus, there was no significant common method bias.

### 3.2. Descriptive statistics

A total of 4,289 older people participated in the survey. The mean age was (*68.82 ± 6.315*) years old, of which 85.6% were younger than 75. The proportion of men (*49.1%*) and women (*50.9%*) is balanced. Overall, the incidence of falls among older people within 3 months was 9.47%. The incidence of falls under 75 years old was 9.23%, and the incidence of falls above 75 years old was 10.88%. Plus, the incidence of falls in older men was 9.03%, and in older women was 9.89%. Within 3 months, the rate of multiple falls was 2.80% among older people. Among those younger than 75 years old, the rate of multiple falls was 31.86, and 17.91% occurred among those older than 75 years old. Among men, the rate of multiple falls was 2.99%, accounting for 33.16% of total falls. For women, the rate of multiple falls was 2.61%, accounting for 26.39% of total falls. Moreover, the big five personality (extraversion, agreeableness, conscientiousness, neuroticism, and openness) scores were (*6.48 ± 1.505*), (*7.00 ± 1.409*), (*7.13 ± 1.525*), (*5.90 ± 1.354*), and (*6.31 ± 1.426*).

Table 1 shows participants’ characteristics and the corresponding distributions of the loneliness scores. Overall, the total loneliness score was (*4.45 ± 1.547*). According to the results, there was a significant difference in age, marital status, education level, residence, solitariness, and fall. According to the *post hoc* test, the loneliness scores of the married were lower than the unmarried, the divorced, and the widowed (*p < 0.001*). The loneliness scores of older people with multiple falls were higher than those with a single fall (*p < 0.001*) and with no fall (*p < 0.001*).

**Table 1:** Loneliness scores among older people with different characteristics (*n* = 4,289).

	Number (%)	Loneliness	*t/F* value	Value of *p*
*Age (years)*			*−3.058*	*0.002*
≤ 75	3,673 (85.6)	4.42 ± 1.523		
> 75	616 (14.4)	4.63 ± 1.671		
*Gender*			*−1.514*	*0.130*
Men	2,105 (49.1)	4.42 ± 1.527		
Women	2,184 (50.9)	4.49 ± 1.565		
*Marital status*			*27.433*	*<0.001*
Unmarried	122 (2.8)	4.98 ± 1.674		
Married	3,576 (83.4)	4.36 ± 1.496		
Divorced	103 (2.4)	4.91 ± 1.652		
Widowed	488 (11.4)	4.92 ± 1.722		
*Education level*			*3.309*	*0.002*
Primary and below	2047 (47.7)	4.53 ± 1.529		
Above primary	2,242 (52.3)	4.38 ± 1.559		
*Monthly family income (¥)*			*0.543*	*0.653*
≤3,000	1881 (43.8)	4.44 ± 1.559		
3,001 ~ 6,000	1760 (41.1)	4.45 ± 1.520		
6,001 ~ 9,000	375 (8.7)	4.55 ± 1.498		
≥9,001	273 (6.4)	4.44 ± 1.695		
*Residence*			*4.646*	*<0.001*
Rural	1919 (44.7)	4.57 ± 1.549		
Urban	2,370 (55.3)	4.35 ± 1.538		
*Solitary*			*−9.712*	*<0.001*
No	3,658 (85.3)	4.36 ± 1.487		
Yes	631 (14.7)	5.00 ± 1.757		
*Fall*			*107.184*	*<0.001*
No	3,883 (90.53)	4.34 ± 1.488		
A single fall	286 (6.67)	5.37 ± 1.670		
Multiple falls	120 (2.80)	5.75 ± 1.788		

### 3.3. Correlation analysis

Between the big five personality traits and fall, the correlation analysis presented that fall was negatively correlated with agreeableness (*r = −0.069*, *p < 0.001*) and conscientiousness (*r = −0.068*, *p < 0.001*); loneliness was positively correlated with neuroticism (*r = 0.085*, *p < 0.001*) and openness (*r = 0.039*, *p = 0.011*); fall was not correlated with extraversion (*r = −0.015*, *p = 0.313*).

Between the big five personality traits and loneliness scores, the correlation analysis presented that loneliness was negatively correlated with extraversion (*r = −0.130*, *p < 0.001*), agreeableness (*r = −0.192*, *p < 0.001*), and conscientiousness (*r = −0.181*, *p < 0.001*); loneliness was positively correlated with neuroticism (*r = 0.170*, *p < 0.001*); loneliness was not correlated with openness (*r = −0.029*, *p = 0.058*).

### 3.4. Multiple linear regression analysis

Multiple linear regression analysis was performed with loneliness as the dependent variable and agreeableness, conscientiousness, and neuroticism as mediating variables and fall as the independent variables. While age, marital status, education level, residence, and solitariness were controlling variables, as shown in Table 2, agreeableness, conscientiousness, neuroticism, and fall were important influence risks for loneliness. After agreeableness, conscientiousness, neuroticism, and fall entered the model, *△R^2^* increased by 5.8%. They were statistically significant with agreeableness (*β = −0.124, p<0.001*), conscientiousness (*β = −0.113*, *p < 0.001*), and neuroticism (*β = 0.170*, *p < 0.001*). In the meantime, the regression coefficient for fall decreased from 0.772 to 0.661 (*p < 0.001*). There was a mediating effect with agreeableness, conscientiousness, and neuroticism.

**Table 2:** Multiple linear regression (*n* = 4,289).

	Model 1		Model 2		Model 3	
	*β*	*p* value	*β*	*p* value	*β*	*p* value
(Constant)	3.988	*<0.001*	3.979	*<0.001*	4.494	*<0.001*
Age	0.158	0.019	0.149	0.024	0.199	0.002
Marital status	0.121	0.001	0.113	0.002	0.143	*<0.001*
Education level	0.031	0.345	−0.011	0.738	0.014	0.650
Residence	−0.216	*<0.001*	−0.167	*<0.001*	−0.136	0.003
Solitary	0.552	*<0.001*	0.492	*<0.001*	0.386	*<0.001*
Fall			0.772	*<0.001*	0.661	*<0.001*
Agreeableness					−0.124	*<0.001*
Conscientiousness					−0.113	*<0.001*
Neuroticism					0.170	*<0.001*
*F* value	26.616	*<0.001*	53.934	*<0.001*	69.994	*<0.001*
*R* ^2^	0.030	*<0.001*	0.070	*<0.001*	0.128	*<0.001*
*△R* ^2^	0.030	*<0.001*	0.040	*<0.001*	0.058	*<0.001*

### 3.5. Mediation model analysis

In this study, fall was set as the independent variable (*X*). When no fall was the reference group, *X*1 represented a single fall, and *X*2 represented multiple falls. Agreeableness (M1), conscientiousness (M2), and neuroticism (M3) were set as the mediating roles. Loneliness was set as the dependent variable (Y). As shown in Table 3, the bootstrap’s 95% CI of agreeableness, conscientiousness, and neuroticism did not overlap the zero; they had a significant mediating effect between fall and loneliness.

**Table 3:** The mediating effect between fall and loneliness through personality traits (*n* = 4,289).

Effect	Estimate	Boot SE	Bootstrap 95%CI	
			Low	High
Total effect (X1 → Y)	1.026	0.093	0.845	1.208
Total effect (X2 → Y)	1.406	0.140	1.132	1.680
X1 → M1 → Y	0.035^#^	0.012	0.014	0.060
X2 → M1 → Y	0.055^#^	0.020	0.019	0.095
X1 → M2 → Y	0.022^#^	0.012	0.001	0.046
X2 → M2 → Y	0.071^#^	0.020	0.034	0.111
X1 → M3 → Y	0.057^#^	0.016	0.027	0.091
X2 → M3 → Y	0.113^#^	0.026	0.065	0.169

# The mediating effect was significant.

## 4. Discussion

In this study, for loneliness among older people, there are significant differences in age, marital status, education level, residence, solitariness, and fall. Many studies showed that age was significantly positively correlated with loneliness (35, 36). People with older subjective ages might not be able to benefit as much from close social relationships as those with younger subjective ages in relieving loneliness (36). Loneliness among the married was lower than among the unmarried, the divorced, and the widowed. When children grew up, they could spend less time caring for their parents. Older people will become more dependent on their spouses, who support each other. They could communicate and interact with each other to create a good family atmosphere. This can relieve loneliness to some extent (37–40). With regard to education level, this study implied that lower levels of loneliness showed up in those with a higher education level, which was consistent with other studies (41, 42). In general, older people with a higher level of education are better at attending affairs and benefitting from more social resources to enhance their lives, which tends to make them experience less loneliness (42). In our study, older people living in urban areas felt significantly less lonely than those living in rural areas. In China, rapid economic development led to a growing number of young people leaving villages to find employment elsewhere, leaving their parents to live alone as empty-nest older people (42). Compared to their rural counterparts, older people who live in urban areas have greater access to healthcare services, technology, and facilities. By taking advantage of these resources, they have more opportunities to network with others, participate in social activities, volunteer, and attend senior colleges (41). Solitariness is a significant risk factor for loneliness among older people all the time (42, 43). As the old saying goes, “The more children, the more happiness.” Frequent contact and communication with family were critical in preventing and relieving loneliness (42). Older people who have few or poor interpersonal relationships could experience more loneliness. Spouses and adult children would provide older people with social and emotional support to relieve their loneliness (44).

We found that falls, only when they occurred one time, increased the loneliness of older people, which was consistent with other studies (13, 15). It was reported that the fallen older people had a significant decrease in activity as a result of the fear of falling, which led to increased loneliness (12). In the meantime, the higher level of loneliness will increase the fear of falls to a greater extent, which will increase the risk of falls (45). This could lead to a vicious cycle. Falls may also increase the burden of caregivers, especially when they take care of people with complex needs (11, 46). According to these reference studies, common conclusions included activity restrictions for managing the fear of falls and preventing falling (46–48). After a fall, older people would reduce social participation actively and caregivers would restrict activities, which increases dependence on caregivers further. In this situation, with fewer social interactions, loneliness will increase.

The current study showed agreeableness, conscientiousness, and neuroticism were meditating factors between falls and loneliness. There was a negative correlation between agreeableness and loneliness, which was consistent with another study (23). Older people with higher agreeableness often have a more positive attitude and trust others more (18). When they fall, they prefer to ask for social support and others’ help. That could relieve the loneliness effectively. There was a negative correlation between conscientiousness and loneliness. Higher conscientiousness often represents the ability to be prudent, responsible, and self-controlled (18). A study indicated that higher conscientiousness was accompanied by higher adherence to adapting to healthy behaviors (49). Therefore, older people with higher conscientiousness would pay more attention to their health and actively prevent falls. Although they fall, they often address it more actively rather than closing themselves off. There was a positive correlation between neuroticism and loneliness, which was consistent with other studies (23, 50). Older people with higher neuroticism are more sensitive to stressful events, which lead to emotional distress or even disorder. They are often unable to cope effectively with pressure and tend to close themselves off (18). When they fall, whether injured or not, they blame themselves and gradually lose contact and restrict activities. As a result, they will experience social isolation and increased loneliness (11, 12).

Although we have found that agreeableness, conscientiousness, and neuroticism were meditating factors between fall and loneliness, the mediating effect coefficient was a bit small. Therefore, we only explored the statistical significance. On the one hand, each big five personality trait was weekly correlated with falls and loneliness (/*r*/ *< 0.30*, *p < 0.05*) in this study. This result only implies a correlation between the three. If we want to explore the relationship and interplay, we should consider the longer-term changes because the big five personality traits remain stable over relatively short periods of time (51). On the other hand, due to many demographic factors affecting loneliness (3) and the complex relationship between the big five personality traits and loneliness (23, 24), we need to exclude disruptions of relevant factors and make research to verify practical values in the future. In the meantime, it has been proven that interventions for social isolation made less effort to relieve loneliness (52). We also explore the complex multi-factor between fall and loneliness. According to recent studies, when we make interventions for older people who have higher fall risks or have experienced falls, it is critical to consider the influence of the Big Five personality traits (22, 24, 53).

## 5. Highlights and limitations

Older people who have experienced falls are more likely to feel lonely. At present, although the relationships among the variables of fall, loneliness, and personality trait have been examined separately, there was a lack of studies to explore the role of personality traits between falls and loneliness. This study indicated that agreeableness, conscientiousness, and neuroticism were significant mediating effects between falls and loneliness. Moreover, it was reported that interventions for social isolation made less effort to relieve loneliness. Therefore, for older people with different personality traits, in the future, interventions could make some adjustments according to distinctions between different personalities.

There were still some limitations that could be improved in future studies. First, self-report questionnaires were used in our study, and the ultra-short measure might be inadequate. Hence, the results of the questionnaire might be affected by participants’ subjective experiences and limited by insufficient assessment. Second, our data were collected during the summer, when older people responded to the questionnaire by their memories, leading to recall bias. Most of the older people were younger than 75 years old, which had an impact on the results. Third, although this study indicated that agreeableness, conscientiousness, and neuroticism had significant mediating effects between falls and loneliness, the mediating effect coefficient was a bit small, partly due to the large sample size. Because the cross-sectional study did not allow for testing the temporal precedence of mediators and causality, in the future, research should explore practical applications. Finally, although our data were from the national level, the distribution of data on older people remained uneven in different regions. Therefore, the conclusions could only reflect certain issues from the side.

## 6. Conclusion

In this study, we found that there are significant differences in age, marital status, education level, residence, solitariness, and fall in relation to loneliness among older people. Falls, especially when they occurred one time increase the loneliness level of older people. This study implied that agreeableness, conscientiousness, and neuroticism were meditating factors between falls and loneliness. In the future, we should consider the big five personality traits more to understand loneliness and offer older people interventions to reduce their loneliness.

## Data availability statement

The raw data supporting the conclusions of this article will be made available by the authors, without undue reservation.

## Ethics statement

Written informed consent was obtained from the individual(s) for the publication of any potentially identifiable images or data included in this article.

## Author contributions

LY, WY, and ZL: study design. LY, WY, DY, RH, and LJ: data collection. LY, DY, RH, and LJ: data analysis and article revision. LY: article writing. WY and ZL: article guide. All authors contributed to the article and approved the submitted version.

## Funding

This study was supported by the Hunan Natural Science Foundation (2021JJ70068).

## Conflict of interest

The authors declare that the research was conducted in the absence of any commercial or financial relationships that could be construed as a potential conflict of interest.

## Publisher’s note

All claims expressed in this article are solely those of the authors and do not necessarily represent those of their affiliated organizations, or those of the publisher, the editors and the reviewers. Any product that may be evaluated in this article, or claim that may be made by its manufacturer, is not guaranteed or endorsed by the publisher.
